# The Effect of Seed-Borne Fungi and *Epichloë* Endophyte on Seed Germination and Biomass of *Elymus sibiricus*

**DOI:** 10.3389/fmicb.2017.02488

**Published:** 2017-12-15

**Authors:** Xiu-Zhang Li, Mei-Ling Song, Xiang Yao, Qing Chai, Wayne R. Simpson, Chun-Jie Li, Zhi-Biao Nan

**Affiliations:** ^1^State Key Laboratory of Grassland Agro-Ecosystems, Key Laboratory of Grassland Livestock Industry Innovation, Ministry of Agriculture, College of Pastoral Agriculture Science and Technology, Lanzhou University, Lanzhou, China; ^2^AgResearch Grasslands Research Centre, Tennent Drive, Palmerston North, New Zealand

**Keywords:** *Epichloë* endophyte, *Elymus sibiricus*, seed-borne fungi, seed germination, *Elymus tangutorum*, *Achnatherum inebrians*, seedling, dry weight

## Abstract

The interactive effects of asexual *Epichloë* (formerly known as *Neotyphodium*) endophytes isolated from *Hordeum brevisubulatum, Elymus tangutorum* and *Achnatherum inebrians*, and seed-borne fungi on *Elymus sibiricus* seeds, were determined by an *in vitro* study using supernatants from liquid cultures of the endophyte strains. In an 8 week greenhouse study, the effects on the seedlings growth was measured. The *in vitro* study was carried out with the seed-borne fungi *Alternaria alternata, Bipolaris sorokiniana, Fusarium avenaceum*, and a *Fusarium* sp. isolated from *E. sibiricus*. Different concentrations and combinations of the liquid cultures of endophytic fungi enhanced the interim germination, germination rate, length of coleoptile and radicle, and seedling dry weight of *E. sibiricus* under stress from seed-borne fungi. In the greenhouse study, different concentrations of the supernatant of the endophytes from *H. brevisubulatum* and *E. tangutorum* but not *A*. *inebrians*, signficantly (*P* < 0.05) enhanced *E. sibiricus* seed germination. There was no significant (*P* > 0.05) increase of the tiller numbers after 2 weeks. However, later on, there were significant (*P* < 0.05) increases in tiller number (4–8 weeks), seedling height (2–8 weeks) and dry weight (2–8 weeks). The application of *Epichloë* endophyte culture supernatants was an effective strategy to improve seed germination and growth under greenhouse conditions.

## Introduction

Endophytic fungal associations with grasses are very common, and the most intensively studied are those between ascomycete fungi and temperate grasses, in particular those involving asexual endophytes of the genus *Epichloë* (Schardl, 2001; Schardl et al., 2004). Asexual or anamorph-typified *Epichloë* have a common origin with the sexual *Epichloë* or teleomorph-typified species (Kuldau et al., 1997; van Zijll de Jong et al., 2011; Leuchtmann et al., 2014). Teleomorph-typified *Epichloë* species are sexually reproducing and cause a condition known as “choke” in grasses, whereby the fungal stromata formed during sexual reproduction leads to reduced flower and seed production (Schardl et al., 2004). The host range of symbiotic fungal endophytes has been described in cool-season grasses (Leuchtmann, 1993; Scott, 2001). Fungal endophytes are of increasing interest due to a growing list of benefits that they can confer on their hosts, including both abiotic and biotic factors such as tolerance to drought (Malinowski and Belesky, 2000; Clay and Schardl, 2002; Hahn et al., 2008), resistance to insects, nematodes and other herbivorous attacks (Omacini et al., 2001; Schardl et al., 2004; Schardl, 2009; Zhang et al., 2012) including bird deterrence (Pennell et al., 2010; Pennell and Rolston, 2012).

Besides that, *Epichloë* endophytes can increase tolerance to pathogenic fungi, although the deployment of *Epichloë* as agents for the biological control of diseases has shown mixed results (Kuldau and Bacon, 2008). *in vitro* suppression of plant pathogens by endophytic fungi has been demonstrated (White and Cole, 1985; Holzmann-Wirth et al., 2000), there is some evidence showing that colony growth of plant-pathogenic fungi is inhibited by *Epichloë* endophytes (Christensen and Latch, 1991; Christensen, 1996; Wäli et al., 2006) and that disease tolerance or resistance can be imparted by *Epichloë* species (Li et al., 2007b; Tian et al., 2008; Porras-Alfaro and Bayman, 2010). The name *Epichloë gansuensis* (*Neotyphodium gansuense*) was proposed by Chunjie Li and Zhibiao Nan (Li et al., 2004; Leuchtmann et al., 2014) for an endophytic fungus symbiotic with *A. inebrians* from Gansu, China. Dual-culture testing and inoculation of detached leaves have shown that *E. gansuensis* can inhibit growth and disease lesion development of some fungal pathogens (Li et al., 2007a).

At present, although many of the mechanisms of the interaction between *Epichloë* and fungal pathogens are not clear, it is reported that several pathogenic fungi are controlled to some level by endophyte infection *in vitro*: *Alternaria alternata, A. triticina, Bipolaris sorokiniana, Cladosporium* spp. including *C. cladosporioides, Curvularia* spp. including *C. lunata, Drechslera erythrospila, Fusarium acuminatum, Phomopsis* spp., *Rhizoctonia cerealis*, and *R. zeae* (White and Cole, 1985; Gwinn and Bernard, 1988; Holzmann-Wirth et al., 2000; Li et al., 2007b; Xie et al., 2008). Compared with un-infected grasses of *Agropyron cristatum, Elymus cylindricus*, and *Festuca rubra, Epichloë* endophyte can reduce the numbers of *Alternaria, Cladosporium*, and *Fusarium* species on leaves of host grasses (Nan and Li, 2000). Vignale et al. report that *E. pampeana* (*N. pampeanum*) and *E. tembladerae* (*N. tembladerae*) can protect their host plant *Bromus auleticus* against the pathogenic fungus *Ustilago bullata* (Vignale et al., 2013). Other studies have demonstrated inhibitory effects *in vivo* against *Ascochyta leptospora, F. avenaceum, F. chlamydosporum, F. culmorum, F. oxysporum, F. solani, Gliocladium roseum, Laetisaria fuciformis*, and *Sclerotinia homeocarpa* (Bonos et al., 2005; Clarke et al., 2006; Li et al., 2007b; Tian et al., 2008).

*Elymus sibiricus* (Siberian wildrye) is a perennial, caespitose grass, widely distributed around the world (Ma et al., 2012). It usually grows on arid or semiarid mountain or valley grasslands at altitudes from 1,000 to 4,000 m in northwestern China. It has also played an important role in native grassland restoration on the Qinghai-Tibet Plateau of China as a pioneer grass species (Ma et al., 2012). *E. sibiricus* usually serves as an important forage grass, and has been widely employed in establishing sown grasslands to develop stock raising, due to its strong adaptability, excellent tolerance to drought and cold, high crude protein content, and good palatability (Yan et al., 2007). However, the pathogenic fungus of seed-borne is important factor to limit the host *E. sibiricus* germination and seedling growth (Li et al., 2007b).

Presently much of the research involving *Epichloë* is concentrated on the relationship with the host grass, secondary metabolites, interaction mechanisms, taxonomy and ecology. Here we examine *Epichloë* endophytes isolated from three species of grass and seed-borne fungi isolated from *E. sibiricus*. The effect of endophytic liquid medium exudate on *E. sibiricus* germination under a seed-borne fungus burden is examined to provide a theoretical basis for the rational use of *Epichloë* endophytes in the field.

## Materials and methods

### *Epichloë* endophyte biological material

*Epichloë gansuensis* (*N. gansuense*) (CBS 119808, ATCC-MYA-3669) was isolated from stems of *A. inebrians* from Sunan, Gansu Province, China (Li et al., 2007b). *Epichloë/Neotyphodium* spp. isolated from *H. brevisubulatum* and *E. tangutorum* were marked as Eb and Et, and the *Epichloë gansuensis* was marked as Eg. The *H. brevisubulatum* and *E. tangutorum* samples were collected from Linze (E:102°54′, N:37°29′; 1,450 m) and Lanzhou (E103°56′, N36°01′; 1,714 m), Gansu Province, China in 2012 (Song and Nan, 2015; Song et al., 2015).

A 4 mm diam plug of 1 week-old endophytic fungus grown on potato dextrose agar (PDA) was used to inoculate 150 mL flasks of potato dextrose broth (PDB) nutrient medium, with 3 repetitions of each strain. Four weeks later, the broth was filtered and centrifuged. The filtrate was diluted to 50 and 25%. The sample of endophytic fungi was marked as Eb01, Eb02, Eb03, Et01, Et02, Et03, Eg01, Eg02 and Eg03, 01 is 25% diluted, 02 is 50% and 03 is undiluted.

### *E. sibiricus* sample and the seed-borne fungi

The *E. sibiricus* seed sample was collected from from Sunan (E99°38′, N38°50′; 2,233 m), Gansu Province, China. Ten seeds were inoculated to each of 10 Petri plates (9 cm diameter) containing PDA, then incubated at 22 ± 1°C in the dark. Observation was made of fungal colony growth on the seed, colonies were picked off to clean PDA plates, and subsequently identified. The 4 fungal strains use in this test were identified as *A. alternaria, B. sorokiniana, F. avenaceum* along with an unidentified *Fusarium* species. Ten replicates of each strain were cultured for 1 week on PDA.

Sterile water was added to the cultures and a spore suspension produced. The spore suspension was micropore filtered. Conidial concentration was determined using a blood count plate and suspensions adjusted to spore concentrations no <10^6^/mL. Adjusted spore suspensions were maintained at 4°C in conical flasks. All isolates of *Epichloë* spp. and fungal pathogens were deposited at the Mycological Herbarium of Lanzhou University.

### Effects of the endophytic fungi supernatant on germination and seedling growth under seed-borne fungi stress

Seeds were surface sterilized in 75% ethanol (v/v) for 5 min and 5% sodium hypochlorite for 10 min, then washed with sterile water 3 times. The seeds were then placed into a centrifuge tube with 15 ml of the endophytic fungi supernatant and imbibed for 12 h under axenic conditions. The control was a centrifuge tube containing the same amount of sterilized water. Seed was incubated in the dark with ventilation. Incubate seed is dry, then sow on two layers of sterile filter paper in a 90 mm glass Petri dish with 5 ml of spore suspension of seed-borne fungi. Each treatment consisted of 4 replicates, for a total of 200 seeds. According to the method of ISTA (1996), all of the seeds were incubated in a growth chamber (25 ± 1°C, 24 h illumination), with sterile distilled water (5 mL) added to each Petri plate every 2 days using a disposable syringe. Germination potential was noted on day 6, percentage of germination on day 12. After 12 day, the radicle length (RL), coleoptile length (CL) and dry weight were measured. The dry weight was obtained after drying at 75°C until a constant weight was recorded (to 0.0001 g) with an electronic balance. The radicle length (RL) and coleoptile length (CL) were measured by Vernier calipers. The above characteristics were calculated by the formula: Interim germination = (the number of germinated seeds at 6 day/total number of seed) × 100%; Germination rate (GR) = (the number of germinated seeds at 12 day/total number of seed) × 100%; Vigor index (VI) = (the number of germinated seeds at 6 day/6^*^length of radicle) × 100%.

### Effects of the endophytic fungi supernatant on plant growth under greenhouse conditions

Uniform, plump seeds were selected and soaked in the Eb03, Et03, and Et02 for 12 h under axenic condition, from this, three supernatant samples were chosen because of they have the effect with high germination rate of the seed. The control consisted of a centrifuge tube containing the same amount of sterilized water. The seed was incubated in the dark with ventilation. Seed was dried and sown into plastic pots (12 cm diameter × 10 cm depth) containing sterile soil (Junzilan, Lanzhou, China), watered as needed, and grown under controlled greenhouse conditions (22/18°C, day/night; sunlight; 65% relative humidity. Each treatment was repeated 5 times independently. Plant height and tiller number were recorded every 2 weeks for 8 weeks. Seedling biomass was harvested after 8 weeks, dried at 75°C and weighted until a constant was recorded.

### Statistical analysis

The data were analyzed for variance (ANOVA) and least significant difference (LSD) using SPSS. 19.0 software (SPSS Inc., Chicago, IL, USA).

## Results

### Effects of the endophytic fungi supernatant on germination and seedling growth under seed-borne fungi stress

Exudates of the 9 endophytic fungi in supernatant when applied to seed were able to enhance interim germination under seed-borne fungi stress, the seed germination was differs under different endophytic fungi sample (Table 1). The seed germination is significantly (*P* < 005) higher when treated with Et supernatant compared to the treatments with Eb and Eg which resulted in higher germination than the (CK) control. For different species of seed-borne fungi, the effect of the application of endophytic fungal supernatant on interim seed germination differed. Across the range of *Epichloë* supernatant examined interim germination of seed exposed to seed-borne fungi *A. alternaria, B. sorokiniana, F. avenaceum*, and *Fusarium* sp. is 21.47–39.13% (mean 29.30%), 18.07–39.93% (mean 27.80%), 13.03–28.60% (mean 19.44%), and 13.77–33.70% (mean 21.47%), respectively. For most strains, as the endophytic fungi supernatant dilution was reduced, the interim germination of seed under seed-borne fungi stress increased. However, the seed germination rate decreased with the increase of concentration of endophytic fungi treated by Eg under all of the seed-borne fungi and Eb03 under *Fusarium* sp.

**Table 1:** Interim Germination (%) of *Elymus sibiricus* under seed-borne fungi stress resulting from effects of the *Epichloë* endophyte.

	***A. alternaria***	***B. sorokiniana***	***F. avenaceum***	***Fusarium* sp**.
CK	22.50 ± 0.96Ea	19.50 ± 2.87Da	11.50 ± 0.96Eb	23.00 ± 1.91Ea
Eb01	26.00 ± 0.82Db	27.50 ± 2.06Cb	17.50 ± 1.71Cc	35.00 ± 3.42Ca
Eb02	31.50 ± 0.50Cb	28.50 ± 1.89Cbc	23.00 ± 1.73Bc	46.00 ± 3.46Ba
Eb03	34.50 ± 0.50Bb	30.00 ± 0.82Cb	24.50 ± 1.26Bc	49.00 ± 2.52Ba
Et01	26.00 ± 0.82Db	25.50 ± 1.26Cb	18.00 ± 0.82Cc	36.00 ± 1.63Ca
Et02	36.50 ± 1.71Bb	35.50 ± 1.26Bb	24.00 ± 0.82Bc	48.00 ± 1.63Ba
Et03	39.50 ± 0.96Ab	40.00 ± 0.82Ab	28.50 ± 1.26Ac	57.00 ± 2.52Aa
Eg01	30.00 ± 0.82Ca	28.50 ± 1.26Ca	16.50 ± 1.26CDb	33.00 ± 2.52CDa
Eg02	26.50 ± 0.96Db	26.00 ± 0.82Cb	17.50 ± 1.26Cb	35.00 ± 2.52Ca
Eg03	21.50 ± 0.96Eb	18.00 ± 0.82Dbc	13.50 ± 1.26Dc	27.00 ± 2.52Da

*Values are mean (±SE) of four independent replications with 50 seeds for each replication at 6 days. Significant differences at the 0.05 level in the same column are indicated by different letters A, B, C, D, E, and in the same row with a, b, c. 01 is 25% diluted of Epichloë endophyte, 02 is 50% and 03 is undiluted*.

The germination rate of the *E. sibiricus* seeds imbibed in endophytic fungi supernatant was significantly (*P* < 0.05) higher than the control (Table 2). Germination rate of the seed of *E. sibiricus* under seed-borne fungi stress increased with increasing endophytic fungi supernatant concentration except for Eb and Eg. With the increase of concentration of endophytic fungi supernatant of Eb, the germination rate under seed-borne fungi stress of *B. sorokiniana* and *F. avenaceum* was higher at first then descended as concentration increased. Eg also showed a higher germination rate across all the seed-borne fungi treatments, but as concentration of the supernatant increased germination rate decreased. However, they had no significant difference (*P* > 0.05). The germination rate of the *E. sibiricus* seeds imbibed in Et supernatant was higher than those imbibed in the other supernatants. The germination rate of the *E. sibiricus* seeds imbibed in the exudates of 3 endophytic fungi, Eb, Et, and Eg is 62.00–78.93% (mean 71.53%), 66.67–85.07% (mean 77.79%), and 61.63–76.80% (mean 69.05%), respectively.

**Table 2:** Germination rate (%) of *Elymus sibiricus* under seed-borne fungi stress resulting from effects of the *Epichloë* endophyte.

	***A. alternaria***	***B. sorokiniana***	***F. avenaceum***	***Fusarium* sp**.
CK	60.00 ± 1.41Da	52.00 ± 0.82Gb	46.50 ± 0.96Fc	54.50 ± 0.96Fb
Eb01	69.00 ± 1.29Ca	70.00 ± 0.82Da	69.00 ± 1.29Ca	62.00 ± 0.82Eb
Eb02	78.50 ± 1.26Ba	73.50 ± 0.50Cb	74.50 ± 1.26Bb	70.00 ± 0.82CDc
Eb03	78.50 ± 1.71Ba	72.00 ± 0.82CDb	72.50 ± 1.71Bb	71.00 ± 1.29BCb
Et01	75.50 ± 0.50Ba	71.50 ± 0.50CDb	66.50 ± 0.50CDc	67.50 ± 0.50Dc
Et02	84.00 ± 0.82Aa	81.00 ± 1.29Bb	81.50 ± 0.50Aab	76.50 ± 0.50Ac
Et03	85.50 ± 0.96Aa	84.50 ± 0.96Aa	83.50 ± 0.50Aa	78.50 ± 0.96Ab
Eg01	72.00 ± 0.82Ca	67.50 ± 0.50Eb	61.50 ± 1.26Ec	69.00 ± 1.29Cab
Eg02	76.50 ± 0.50Ba	74.00 ± 0.82Cb	68.50 ± 0.50Cc	73.50 ± 0.96Bb
Eg03	70.00 ± 1.41Ca	65.00 ± 1.29Fb	64.50 ± 1.26Db	68.50 ± 1.26CDab

*Values are mean (±SE) of four independent replications with 50 seeds for each replication at 12 days. Significant differences at the 0.05 level in the same column are indicated by different letters A, B, C, D, E, F, G, and in the same row with a, b, c. 01 is 25% diluted of Epichloë endophyte, 02 is 50% and 03 is undiluted*.

Coleoptile length of *E. sibiricus* under seed-borne fungi stress increased with increasing concentration of most endophytic fungi supernatant with the exception of the Eg exudate (Table 3). The coleoptile length of *E. sibiricus* seedlings generated from seed imbibed in Eg supernatant and exposed to *A. alternaria* and *Fusarium* sp. increased as the concentration of Eg liquid medium increased but then decreased with increasing concentration 3.97, 4.07, 4.03, 3.92, 4.04, and 4.01 cm, however there was no significant difference (*P* > 0.05). The coleoptile length of seedlings generated from seed exposed to the supernatant of the 3 fungal strains were significantly (*P* < 0.05) higher than that of the control. The increase in coleoptile length observed for seedlings generated from endophytesupernatant imbibed seed under seed-borne fungi burden varied. The coleoptile length of seedlings treated by Et02 and Et03 were significantly (*P* < 0.05) higher than the others, with coleoptile lengths of 4.47–4.97 cm (mean 4.70 cm), 4.50–4.91 cm (4.79 cm), respectively.

**Table 3:** Coleoptile length (cm) of *Epichloë* endophyte-infected *Elymus sibiricus* under seed-borne fungi stress.

	***A. alternaria***	***B. sorokiniana***	***F. avenaceum***	***Fusarium* sp**.
CK	3.11 ± 0.03Ea	3.09 ± 0.06Ea	2.87 ± 0.05Fb	3.01 ± 0.03Ea
Eb01	3.59 ± 0.05Da	3.45 ± 0.05Dab	3.53 ± 0.11Ea	3.28 ± 0.05Db
Eb02	4.06 ± 0.06BCa	4.01 ± 0.09BCab	3.80 ± 0.05BCDb	3.97 ± 0.08Bab
Eb03	4.16 ± 0.06Ba	4.09 ± 0.04Bab	3.97 ± 0.03Bb	4.01 ± 0.03Bb
Et01	3.90 ± 0.05Cab	3.94 ± 0.05BCa	3.66 ± 0.05DEb	3.69 ± 0.11Cb
Et02	4.59 ± 0.12Abc	4.78 ± 0.09Aab	4.97 ± 0.02Aa	4.47 ± 0.05Ac
Et03	4.71 ± 0.08Ab	4.91 ± 0.02Aa	5.04 ± 0.06Aa	4.50 ± 0.07Ac
Eg01	3.97 ± 0.03BCa	3.53 ± 0.14Db	3.75 ± 0.04CDab	3.92 ± 0.06Ba
Eg02	4.07 ± 0.04BCa	3.78 ± 0.09Cb	3.91 ± 0.03BCab	4.04 ± 0.05Ba
Eg03	4.03 ± 0.03BCa	3.83 ± 0.08Ca	3.95 ± 0.06Ba	4.01 ± 0.03Ba

*Values are mean (±SE) of four independent replications with 50 seeds for each replication at 12 days. Significant differences at the 0.05 level in the same column are indicated by different letters A, B, C, D, E, and in the same row with a, b, c. 01 is 25% diluted of Epichloë endophyte, 02 is 50% and 03 is undiluted*.

Radicle length of *E. sibiricus* under seed-borne fungi stress increased with increasing concentration of endophytic fungi liquid medium except with Et exudate (Table 4). The radicle length of *E. sibiricus* under *A. alternaria* pressure increased as the concentration of Eg exudate increased but then decreased with increasing exudate concentration however there was no significant difference (*P* > 0.05). The radicle length of *E. sibiricus* treated with Et02 and Et03 were significantly (*P* < 0.05) higher 3.41–3.92 cm (mean 3.71 cm) and 3.86–4.08 cm (mean 3.99 cm), than the others. The radicle length of endophyte exudate treated *E. sibiricus* under seed-borne fungi stress involving *A. alternaria, B. sorokiniana, F. avenaceum*, and *F*. sp. was 2.15–3.33 cm (mean 2.84 cm), 2.42–4.08 cm (mean 3.49 cm) and 2.39–2.76 cm (mean 2.57 cm), respectively, which was higher than the control at 1.92–2.14 cm (mean 2.02 cm).

**Table 4:** Radicle length (cm) of *Elymus sibiricus* under seed-borne fungi stress resulting from effects of the *Epichloë* endophyte.

	***A. alternaria***	***B. sorokiniana***	***F. avenaceum***	***Fusarium* sp**.
CK	2.14 ± 0.04Ca	2.04 ± 0.02Fab	1.92 ± 0.04Eb	1.98 ± 0.04Fb
Eb01	2.26 ± 0.03Cab	2.32 ± 0.06Ea	2.34 ± 0.04Da	2.15 ± 0.03Fb
Eb02	2.93 ± 0.16Ba	3.24 ± 0.10Ba	2.99 ± 0.07Ca	3.17 ± 0.02Ca
Eb03	3.03 ± 0.04Bb	3.33 ± 0.05Ba	3.08 ± 0.02Cb	3.25 ± 0.07Ca
Et01	2.95 ± 0.22Ba	2.92 ± 0.20Ca	2.42 ± 0.06Da	2.76 ± 0.13Da
Et02	3.88 ± 0.15Aa	3.92 ± 0.13Aa	3.41 ± 0.09Bb	3.61 ± 0.02Bab
Et03	4.08 ± 0.04Aa	4.06 ± 0.02Aa	3.86 ± 0.10Aa	3.95 ± 0.06Aa
Eg01	2.74 ± 0.04Ba	2.56 ± 0.06DEb	2.39 ± 0.03Dc	2.42 ± 0.03Ec
Eg02	2.76 ± 0.14Ba	2.67 ± 0.08CDa	2.46 ± 0.05Da	2.50 ± 0.08Ea
Eg03	2.69 ± 0.07Ba	2.71 ± 0.08CDa	2.47 ± 0.04Db	2.53 ± 0.05Eab

*Values are mean (±SE) of four independent replications with 50 seeds for each replication at 12 days. Significant differences at the 0.05 level in the same column are indicated by different letters A, B, C, D, E, F, and in the same row with a, b, c. 01 is 25% diluted of Epichloë endophyte, 02 is 50% and 03 is undiluted*.

Dry weight of *E. sibiricus* seedlings under seed-borne fungi stress increased with increasing concentration of endophytic fungi exudate except for Eg (Table 5). The dry weight of seedlings treated with *A. alternaria* and *F. avenaceum* initially increased with increasing concentration of Eg exudate then decreased. Treatment of *B. sorokiniana* stressed seedlings with Eg resulted in a relative seedling dry weight decrease, initially, followed by an increase with increasing exudate concentration, but with no significant difference (*P* > 0.05).

**Table 5:** Dry weight (10^−2^g) of *Elymus sibiricus* under seed-borne fungi stress resulting from effects of the *Epichloë* endophyte.

	***A. alternaria***	***B. sorokiniana***	***F. avenaceum***	***Fusarium* sp**.
CK	1.95 ± 0.04Ca	1.93 ± 0.03Fab	1.83 ± 0.02Db	1.91 ± 0.03Dab
Eb01	2.12 ± 0.02ABCc	1.98 ± 0.07EFab	1.95 ± 0.05Cb	2.01 ± 0.02BCab
Eb02	2.21 ± 0.01Aa	2.17 ± 0.02BCDa	2.04 ± 0.04Bb	2.08 ± 0.03Bb
Eb03	2.23 ± 0.06Aa	2.20 ± 0.05BCab	2.07 ± 0.02Bb	2.09 ± 0.01Bb
Et01	2.14 ± 0.03ABa	2.09 ± 0.03CDEab	2.02 ± 0.03BCb	2.03 ± 0.04BCab
Et02	2.27 ± 0.06Aa	2.30 ± 0.08ABa	2.16 ± 0.01Aa	2.21 ± 0.02Aa
Et03	2.28 ± 0.11Aa	2.35 ± 0.06Aa	2.18 ± 0.02Aa	2.23 ± 0.02Ba
Eg01	1.98 ± 0.06BCa	2.05 ± 0.04DEFa	2.02 ± 0.03BCa	1.97 ± 0.03CDa
Eg02	2.02 ± 0.02BCa	2.03 ± 0.04DEFa	2.05 ± 0.02Ba	1.99 ± 0.03Ca
Eg03	1.97 ± 0.03Ca	2.04 ± 0.01DEFa	2.03 ± 0.02BCa	2.01 ± 0.03BCa

*Values are mean (±SE) of four independent replications with 50 seeds for each replication at 12 days. Significant differences at the 0.05 level in the same column are indicated by different letters A, B, C, D, E, F, and in the same row with a, b, c. 01 is 25% diluted of Epichloë endophyte, 02 is 50% and 03 is undiluted*.

### Effects of the endophytic fungi liquid medium on plant growth under greenhouse conditions

**Figure 1:**
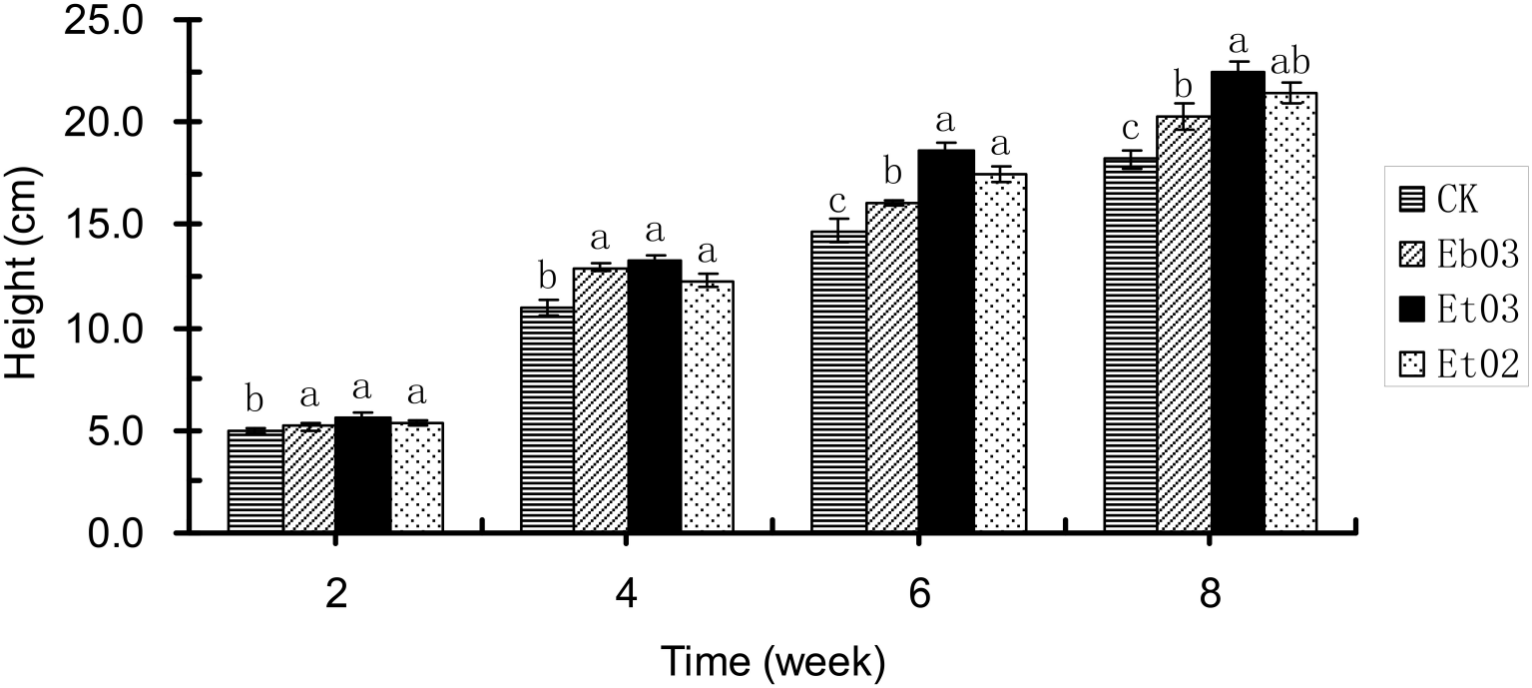
Effects of *Epichloë* endophyte on plant height of *Elymus sibiricus*. Significant differences at the 0.05 level are indicated within a time by different letters above bars. Values are means of five replicates ±SE.

Plant height of *E. sibiricus* treated by the optimal concentration of Eb03, Et03 and Et02 was significantly (*P* < 0.05) higher than the control during 2–8 weeks (Figure 1). The mean plant height of the treatment is 5.4, 12.8, 17.4, and 21.4 cm at 2, 4, 6, and 8 week, respectively. The plant height difference among the 3 treatments was not significant (*P* > 0.05) during 2–4 weeks, from 4 to 8 weeks plants treated with Et03 and Et02 showed no significant (*P* > 0.05) difference, but the difference between plants treated with Et03 and Eb03 was significant (*P* < 0.05). The height of plants treated with Et03 is higher than that of plants subjected to other treatments during 2–8 weeks, and significantly (*P* < 0.05) higher than the control of 4.9, 11.0, 14.7, and 18.2 cm.

**Figure 2:**
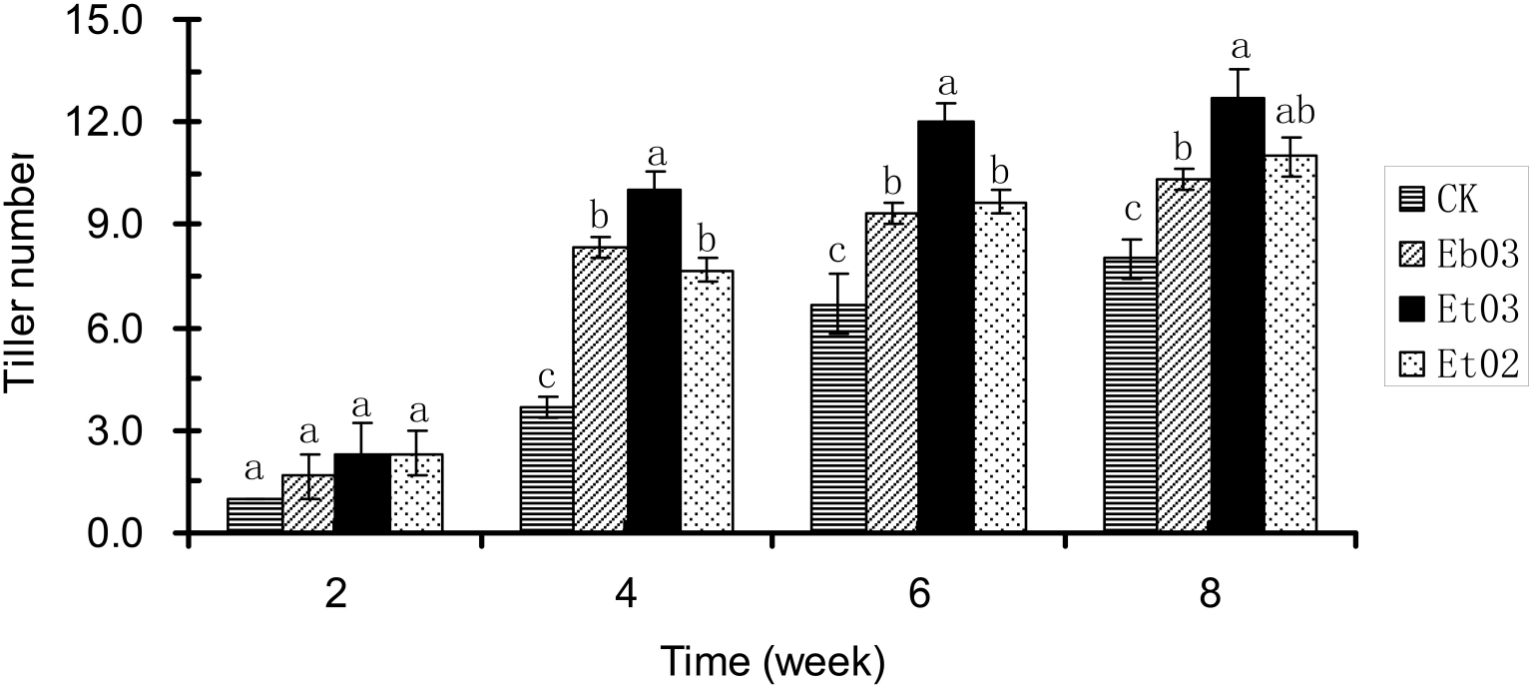
Effects of *Epichloë* endophyte on tiller numbers of *Elymus sibiricus*. Significant differences at the 0.05 level are indicated within a time by different letters above bars. Values are means of five replicates ±SE.

After 2 weeks, the tiller numbers of the treatment and control showed no significant (*P* > 0.05) difference (Figure 2). The tiller number of treated plants was significantly (*P* < 0.05) higher than the control plants during 4–8 weeks. Plants treated with Et03 had more tillers than the other treatments, however this was not significant (*P* > 0.05) with respect to plants treated with Eb03 and Et02. Treatment with endophyte supernatant resulted in large effects on the formation of tillers during the 4–6 week period. The tiller number of treated plants was significantly higher than that of the control plants by 29.17, 58.33, and 37.50%, respectively.

**Figure 3:**
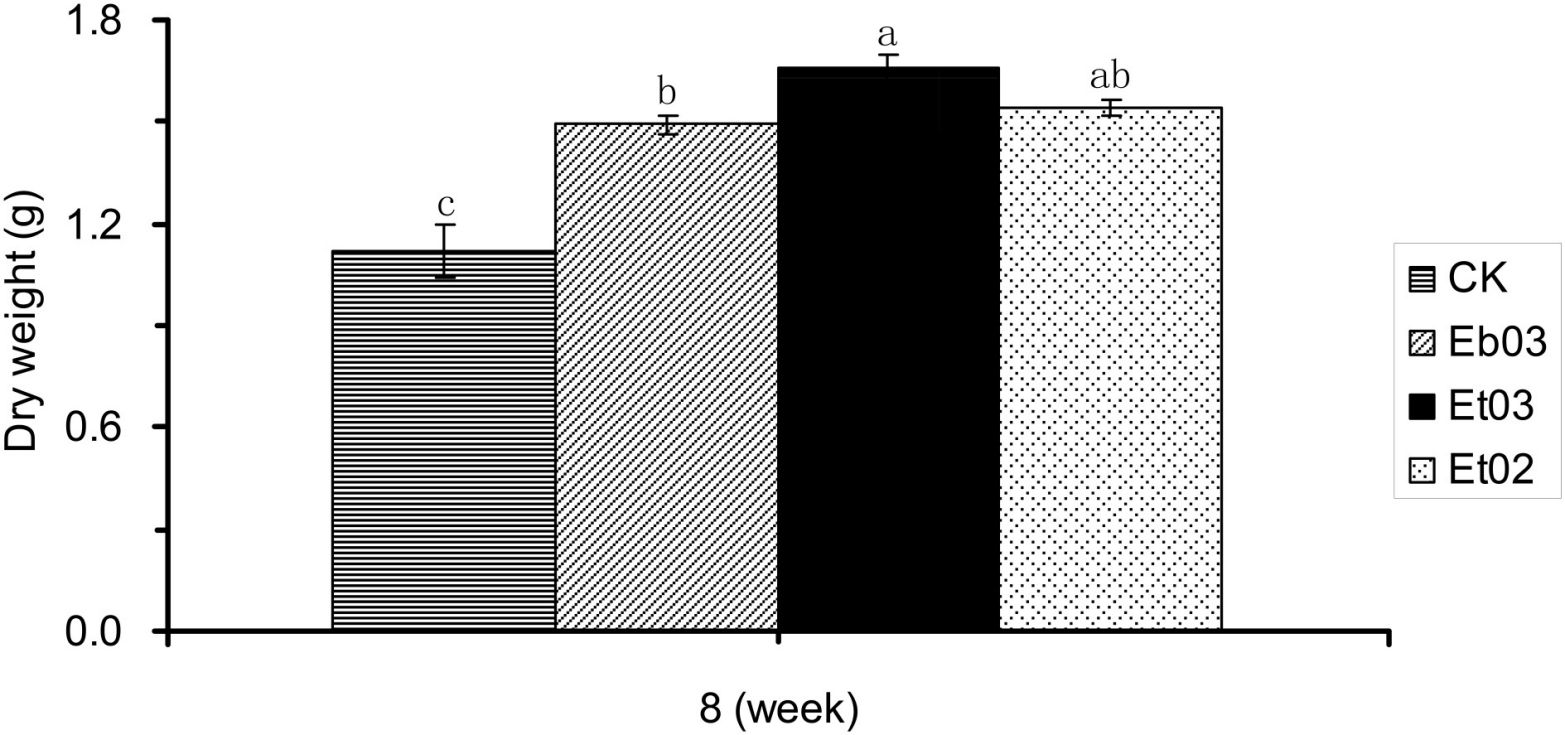
Effects of *Epichloë* endophyte on dry weight of *Elymus sibiricus*. Significant differences at the 0.05 level are indicated within a time by different letters above bars. Values are means of five replicates ±SE.

At the final harvest after 8 weeks, treatment with endophyte supernatant from Eb03, Et03, and Et02 resulted in a significant (*P* < 0.05) increase of total dry weight, higher than the control seedlings by 37.20, 53.63, and 42.43%, respectively (Figure 3). Among the three different treatments, that treated by Et03 was higher than the others, the effect of the other endophytes was not significant (*P* > 0.05).

## Discussion

Biological control of plant diseases is an important effect observed on grasses infected by *Epichloë* endophytes although there are mixed results. It is observed that epiphyllous mycelial nets in some endophyte-grass associations may play a role in defense against pathogens by niche exclusion (Moy et al., 2000). The research methods of endophytic fungi interaction with pathogenic fungi are mainly concentrated in dual-culture on plate, PDB trials, *in vitro* inoculation, vital inoculation and field trial. The present work, to our knowledge, is the first report of the protective effect of *Epichloë* endophytes against seed-borne fungi, and resulting in increased seed germination and plant growth.

Previous research has shown that *Epichloë* can have inhibition activity against pathogenic fungi such as *Alternaria, Bipolaris, Fusarium, Cladosporium, Pythium, Curvularia, Drechslera, Rhizoctonia*, and so on (Holzmann-Wirth et al., 2000; Nan and Li, 2004; Li et al., 2007a; Tian et al., 2008). In this work we found that, compared with controls, the *Epichloë* endophyte increases interim germination, germination rate, coleoptile length, radicle length and dry weight of *E. sibiricus* to varying degrees when plants are under stress from seed-borne fungi such as *A. alternaria, B. sorokiniana, F. avenaceum* and *Fusarium* sp.

Similar results were obtained by New Zealand scientists, which ranged from nil to strong inhibition on growth and conidial germination of grass-pathogenic fungi by *E. festucae var. lolii* (*N. lolii*) and endophytes in *in vitro* tests (Christensen and Latch, 1991; Christensen et al., 1991). A previous study with *E. coenophiala* (*N. coenophialum*) showed effective inhibition of growth of *A. alternata* (White and Cole, 1985); *E. festucae var. lolii* (*N. lolii*) effectively inhibited *Drechslera* spp. (Holzmann-Wirth et al., 2000). Nan and Li (2000) showed that detached leaves of E+ *Elymus cylindricus* had fewer and smaller lesions than those on E– plants inoculated with *A. alternata, F. avenaceum*. These studies addressed antifungal activities of endophyte *in vitro* or on detached leaves of other grass species. That the total lengths of lesions on detached leaves were greater (*P* < 0.05) on E– plants than on E+ plants when inoculated with the plant pathogens *A. alternata, B. sorokiniana, C. lunata, F. acuminatum, F. avenaceum*, and 10 other species of pathogenic fungi. Although differences between E+ and E– were not consistently significant at all sample times (days after inoculation) in detached leaves. The numbers of lesions were greater (*P* < 0.05) and the lesions were larger (*P* < 0.05) on intact E– plants than on intact E+ plants for the pathogens of four pathogens (*A. alternata, B. sorokiniana, Curvularia lunata* and *F. avenaceum*) when living plants were studied (Tian et al., 2008).

This study found that compared with the seeds without soaking with endophytic fungi supernatants, the plant height, tiller and biomass all have different degrees of increase. This is similar to the *Epichloë* endophyte induced improvments in plant height, tillering and biomass of grasses, including *A. inebrians* (Nan and Li, 2000), *F. arundinacea* (Joost, 1995) and *L. perenne* (Clay, 1987). Latch et al. (1985) reported that *L. perenne* infected with *E. festucae* var. *lolii* (*N. lolii*) resulted in dry weights nearly 38% higher than the non-infected *L. perenne*, and the leaf area, number of branches and root dry weight were also significantly (*P* < 0.05) higher than the control. Compared with the un-infected *F. arundinacea, E. coenophiala* (*N. coenophialum*), endophyte significantly (*P* < 0.05) increases the production, performance and the grass coverage by 20–30% 4 months after sowing (Joost, 1995). The endophytic fungi can increase forage yield of 22–55%, the number of tillers 20–45% and the seed weight 26–41% (Clay et al., 1993). Nan and Li (2000) founds that endophytic fungi infection of *H. bogdanii* significantly (*P* < 0.05) promotes the growth of host plants, the plant tiller number increased by 136.8%, herbage yield increased by 33.3% and root dry weight increased by 30%. In filed experiments examining *E. cylindricus*, the endophyte increased the tiller number by 84.5%, the above ground dry weight increased by 278.7% and the tiller weight increased by 105.3%. A potted plant experiment showed that the extracts from E+ *A. inebrians* remarkably enhanced the growth of *F. arundacea, L. perenne* and *P. pratensis* (Yang et al., 2010).

Tillering of grasses is controlled, amongst other things, by indole acetic acid and other plant hormones. Endophytic fungi have the ability to produce indole acetic acid, this might be one of the reasons for the promotion of tillering in infected plants (West and Gwinn, 1993). However, in this study, there were no significant (*P* > 0.05) differences of plant tiller number between plants that were treated with endophyte exudate and untreated controls in the 2 weeks under greenhouse conditions. This absence of effect at the *P* < 0.05 level might be due to low levels of indole acetic acid being produced by the seeding at this early stage of growth.

Based on the results of the germination test presented here, the seed germination decreased with increased culture concentration of the endophytic fungi, similar to the results of Huang et al. (2010) research results. They reported that the water extraction of the E+ *A. inebrians* had significant inhibitory effects on seed germination and seedling growth of *S. capillata* and *P. sphondylodes* using a Petri dish-paper germination method. Inhibition is also seen on the seed germination rate of *L. perenne* and bud length of *P. pratensis* by the same method. But the potted experiment showed that the grass powder of E+ *A. inebrians* accelerated plant growth and initial seedling emergence rates of *L. perenne* and *P. pratensis*. It is speculated that the ergonovine and ergine concentration from *A. inebrians*/*E. gansuensis* (*N. gansuense*) may be responsible for this effect (Yang et al., 2010). Petroski et al. (1990) reported that loline has strong allelopathy to annual ryegrass and alfalfa. The loline produced by *F. arundinacea* has higher allelopathy to its competitors (Malinowski and Belesky, 2000). The suggestion that the inhibitory effects of Eg and Eb on seed germination due to their heigher alkaloid concentrations requires further study.

Here we have demonstrated that fungal culture supernatants can greatly promote germination and subsequent plant growth under seed-borne fungi stress. However, high concentrations can suppress growth, this action appears to be complex. The interactive mechanisms of *Epichloë* endophyte and seed-borne fungi require further study, and the effective range of concentrations of different endophyte exudates needs to be determined. This may eventually provide insight into strategies for the improvement of field performance and stress tolerance in grasses of forage and turf.

## Author contributions

XZ-L designed and performed experiments, analyzed the data and wrote the manuscript. CJ-L designed experiments, polished manuscript, provided reagents and experimental equipment. ML-S, XY, and QC performed experiments and analyzed the data. WS analyzed the data and polished manuscript. ZB-N provided reagents and experimental equipment. All authors reviewed the manuscript.

### Conflict of interest statement

The authors declare that the research was conducted in the absence of any commercial or financial relationships that could be construed as a potential conflict of interest.
